# Morphological Evaluation of Maxillary Premolar Canals in Iranian Population: A Cone-Beam Computed Tomography Study

**DOI:** 10.30476/DENTJODS.2020.82299.1011

**Published:** 2020-09

**Authors:** Bahar Asheghi, Nariman Momtahan, Safoora Sahebi, Maryam Zangoie Booshehri

**Affiliations:** 1 Dept. of Endodontics, School of Dentistry, Shiraz University of Medical Sciences, Shiraz, Iran; 2 Student Research Committee, School of Dentistry, Shiraz University of Medical Sciences, Shiraz, Iran; 3 Dept. of Oral and Maxillofacial Radiology, School of Dentistry, Shahid Sadoughi University of Medical Sciences, Yazd, Iran

**Keywords:** Cone-Beam Computed Tomography, Maxillary premolar, Root morphology, Iranian population

## Abstract

**Statement of the Problem:**

A thorough knowledge of the morphological and anatomical variations of root canal system can help identify all root canals, adequate instrumentation, and consequently leads to a successful endodontic treatment. The knowledge of root morphology can influence the outcome of root canal therapy.

**Purpose:**

The aim of this study was to investigate the morphology of maxillary premolar canals in Iranian population by analyzing cone-beam computed tomography (CBCT) scans.

**Materials and Method:**

This cross-sectional retrospective study consisted of CBCT of 280 patients over 16 years of age. The position, number of roots, and root morphology of maxillary premolars were inspected. The root canal configurations of maxillary premolar teeth were also analyzed according to the Vertucci classification. The statistical analyses wee performed using chi-square test. Significance level was set at *p* < 0.05.

**Results:**

In the present study, of 462 maxillary first premolars, 8 (1.73%) teeth had three roots, 222 (48.05%) teeth had two roots, and 232 (50.22%) were single-rooted. In the second maxillary premolar group, of 400 premolars, 2 (0.5%) teeth had three roots, 34 (8.5%) teeth were two-rooted, and 364 (91%) were single-rooted. The most root canal configurations were type IV (71.64%) and type I (63%) in maxillary first and second premolars, respectively. Among females, single rooted premolars were the most prevalent (56.83%), and among males, two-rooted premolars were the most prevalent (57.61%).

**Conclusion:**

This study provided information about the root canals of maxillary premolar teeth for Iranian subpopulation leading to more optimal diagnosis and treatment planning for the endodontists. According to the findings, the complexity of root canal system and the number of roots were less common in females compared to males.

## Introduction

Success in root canal treatment depends entirely on cleaning and disinfection of all root canals in a teeth [ 1 ]. Inadequate knowledge of the root canal anatomy results in missing and insufficient cleaning of root canals. The complexity of root canal anatomy is the cause of most root canal treatment failures including missing root canals [ 2 ]. Among different groups of teeth, root canal treatment of premolar teeth has always been a challenge for clinicians [ 3 ]. Many studies of different populations have reported an extremely diverse and complex root and canal morphology for the premolar teeth showing additional canals and roots [ 4 , 6 ]. Several studies have reported that maxillary premolars have a highly variable internal canal configuration, which can vary according to race and geographic origin [ 4 , 7 ].

Previous studies have reported a prevalence of 22% to 66% for single rooted, 33% to 84% for two-rooted, and 0 to 6% for three-rooted maxillary first premolar [ 3 , 5 , 7 , 12 ]. Vertucci *et al.* [ 8 ] showed that there is a third root in 5-6% of maxillary first premolars.

Significant anatomical variation has also been observed in the maxillary second premolar. Vertucci, in a research performed on maxillary second premolars, reported a prevalence of 1% for three-rooted second premolars [ 13 ]. The incidence of three canals in maxillary premolars has also been reported to vary from 0% to 10% [ 14 , 15 ]. 

One of the factors that can affect the results of prevalence studies on the morphology of teeth in diverse populations is the various in-vivo and in-vitro methods used to identify root canal morphology in different studies [ 16 , 17 ].

Recently, the application of in-vivo methods such as cone-beam computed tomography (CBCT), that can create a three-dimensional image in the axial, sagittal and coronal dimensions with the precision of canal staining and clearing techniques and without the limitations of conventional radiography such as distortion and superimposition of images, has become increasingly popular [ 18 , 21 ].

In addition, when using CBCT, patients’ demographic data such as age and gender as well as the symmetry of specific morphologies can be verified while this may not be feasible in the in-vitro methods performed on extracted teeth [ 22 ].

Besides the investigation methods, other factors such as sex [ 7 , 23 , 24 ], age [ 25 , 26 ], race, or ethnicity [ 26 ], and the geographical location [ 6 ] can affect the root canal system especially in the premolar teeth and subsequently can influence the morphological complexity of the canal. Therefore, clinicians should be aware of the anatomy of this group of teeth to be able to use appropriate therapeutic techniques to achieve higher success rate in root canal treatment.

Several studies have evaluated the anatomy of maxillary premolars in different Iranian populations [ 27 , 30 ]. Considering the vastness of different races of people in Iran, there is a need to study of number and root morphology of maxillary premolars in the south of Iran. 

Therefore, the aim of this study was to investigate the root and canal morphology of maxillary premolar teeth in relation to sex and position in an Iranian subpopulation in the south of the country by using CBCT from 2015 to 2018.

## Materials and Method

CBCT radiographs of 280 patients referred to Shiraz radiology clinics between 2015 and 2018 were used in this cross-sectional retrospective study. The sample size was determined in line with literature. The CBCT images used in this study were taken for other purposes such as implant planning, prosthesis, surgery, orthodontic and endodontic purposes. Of these images, 862 bilateral maxillary premolars were selected using simple random sampling. The study approval was obtained from Ethics Committee of Shiraz University of Medical Sciences (IR. SUMS.REC.1397.120). 

The CBCTs enrolled in this study have been collected from the patients between 16 and 60 years of age who had intact maxillary premolars with completed root formation and without root canal treatment. Teeth with any signs of filling, external, and internal root canal resorption, periapical lesions, post, crown or calcification were excluded from the study. In addition, the images of teeth with compromised anatomy and poor quality images were withdrawn from the study.

All images were prepared using Promax 3Dmax (PlanMeca®), Helsinki, Finland at 90 kVp, 14 mA, with an exposure time of 15s. These parameters were automatically set by the device according to the patients’ size and weight. A maximum field of view of 10x10cm, voxel size of 150µm, and mode of high definition was used. 

Images were evaluated using Romexis imaging software version 3.8.2 on a 23-inch monitor in a dim light. Using axial, coronal, and sagittal planes, all of the CBCT images were inspected retrospectively by a calibrated endodontist and senior dental student who were trained to interpret images independently, with a two-week interval between the assessments. Prior to the experiment, the investigators graded 50 samples of CBCT images. If any disparity was seen between the opinions, a radiologist to reach a consensus evaluated the images. To assess the intraexaminer reliability, a re-assessment was performed one month following the first session. The data obtained from CBCTs were recorded as follows: 

1. The number of roots from the axial plane based on the classification of Pecora *et al.* [ 31 ]

2. The number of root canals in each root

**Figure 1:**
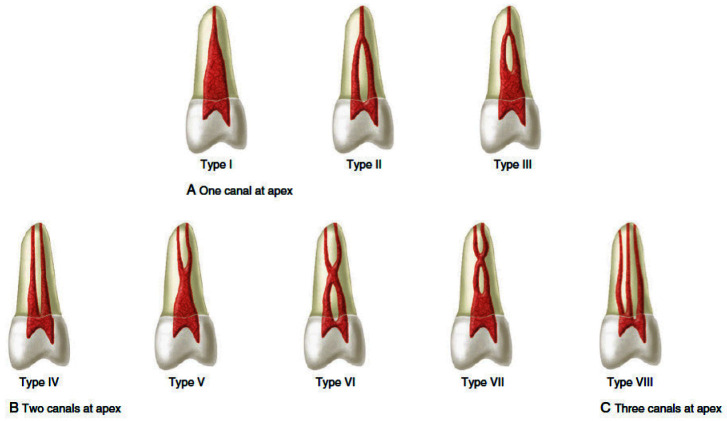
Vertucci classification.

The root canal configuration (based on the Vertucci classification) (Figure 1) [ 32 ] 

3. Position of the teeth in jaws for bilateral symmetry evaluation

4. Patients' gender based on demographic records of CBCTs

### Data Analyses

Data were analyzed using SPSS software version 18 (SPSS Inc, Chicago, IL, USA). Chi-square test was used to study the frequency of various morphologies between different sex and root morphology groups. Quantitative data was described with mean plus standard deviation and qualitative data was presented using frequency (percent). Significance level was set at *p*< 0.05. Differences in tooth position (right or left) were determined by using the X^2^ test and were considered significant if the *p* Value was <.05. Intraexaminer and interexaminer agreements were calculated by using the Cohen kappa coefficient.

## Results

Of 280 CBCT images, 862 bilateral maxillary premolars were included in the study, of which 462 were maxillary first premolar and 400 were maxillary second premolar. In this study, the number of roots recorded for the maxillary first and second premolars has been up to three roots, and the canal configuration of these teeth were classified as type I to type VIII (Table 1).

**Table 1:** Classification of maxillary premolars according to number of roots and type of canal.

	No. of roots	Types of canal configuration								Total
		I (%)	II (%)	III (%)	IV (%)	V (%)	VI (%)	VII (%)	VIII (%)	
Maxillary 1^st^ premolars	One	41(17.67)	70(30.17)	4 (1.72)	110(47.41)	6 (2.59)	1 (0.43)	0	0	232 (50.22%)
	Two	0	0	0	221(99.55)	0	0	0	1 (0.45)	222 (48.05%)
	Three	0	0	0	0	0	0	0	8(100)	8 (1.74%)
	Total	41(8.87)	70(15.15)	4 (0.86)	331(71.64)	6 (1.30)	1 (0.22)	0	9 (1.95)	462
Maxillary 2^nd^ premolars	One	252(69.23)	78(21.43)	3 (0.82)	25 (6.87)	4 (1.10)	2 (0.55)	0	0	364 (91%)
	Two	0	0	0	32(94.12)	0	0	0	2 (5.88)	34 (8.5%)
	Three	0	0	0	0	0	0	0	2 (100)	2 (0.5%)
	Total	252(63.0)	78 (19.5)	3 (0.75)	57(14.25)	4 (1.0)	2 (0.5)	0	4 (1.0)	400

### Maxillary first premolar

#### Number of roots

Of the 462 maxillary first premolars, 232 teeth (50.22%) were single-rooted presenting the highest prevalence in this subpopulation; 222 teeth (48.05%) had two roots, and 8 teeth (1.74%) had three roots. From the two-rooted maxillary first premolars, in 45.95% (n=102) cases, the roots were separated into two independent roots from the floor of pulp chamber, and in 54.05% (n=120) cases, the roots were separated at different levels of the root.

#### Type of roots

Overall, the most common canal configuration for the maxillary first premolar was type IV (n = 331; 71.64%), followed by type II (n = 70; 15.15%) and type I (n = 41; 8.87%). Other types made up 4.33% of all canal configurations, which is a low percentage.

#### Number of apical foramens

The first maxillary premolar with 1, 2 or 3 apical foramens were observed in approximately (n=115; 24.89%), (n= 338; 73.16%), and (n= 9; 1.95%) cases respectively. In the single-rooted maxillary first premolars, 115 teeth (49.57%) had one foramen in root and 117 (50.43%) had two foramens. While all the two-rooted maxillary first premolars had one foramen in each root, there was only one case with two foramens in the buccal root and one foramen in the palatal root. In all three-rooted first premolars, there was one foramen in each root.

### Maxillary Second Premolar

#### Number of roots

**Figure 2:**
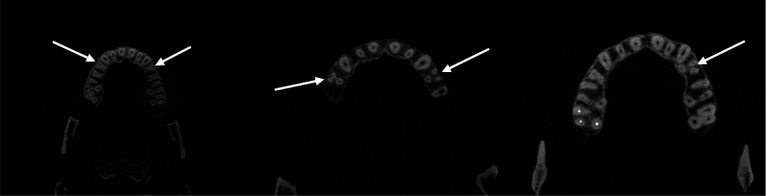
CBCT (Cone-Beam Computed Tomography) in the axial plane revealed single-rooted maxillary first and second premolars (arrows indicate the examined teeth)

Of the 400 maxillary second premolars, the highest prevalence was observed for single roots (n= 364; 91%), followed by two roots (n= 34; 8.5%) and three roots (n= 2; 0.5%). From the two-rooted maxillary second premolars, in 38.24% (n=13) cases, the roots were separated into two independent roots from the floor of pulp chamber, and in 61.76% (n=21) cases, the roots were fused. Figures 2 to [Fig JDS-21-215-g003.tif] [Fig JDS-21-215-g004.tif] 5 show a selection of root numbers detected in different sections.

**Figure 3 JDS-21-215-g003.tif:**
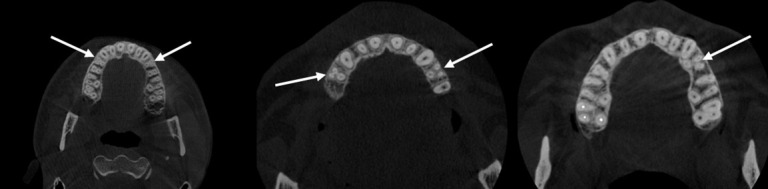
CBCT (Cone-Beam Computed Tomography) in the axial plane revealed three-rooted maxillary first premolars (arrows indicate the examined teeth)

**Figure 4 JDS-21-215-g004.tif:**
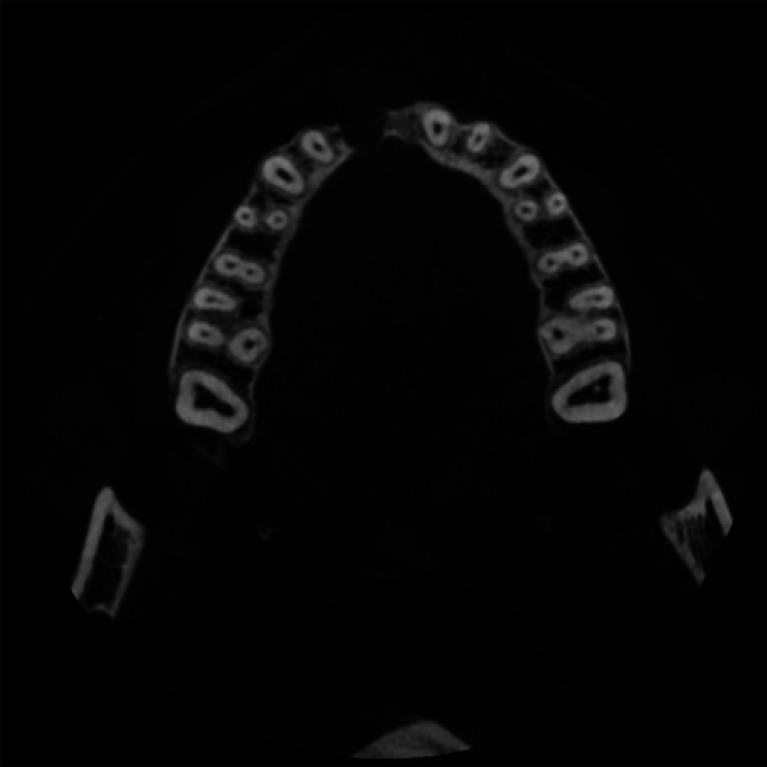
CBCT (Cone-Beam Computed Tomography) in the axial plane revealed two-rooted maxillary first premolars (arrows indicate the examined teeth)

**Figure 5:**
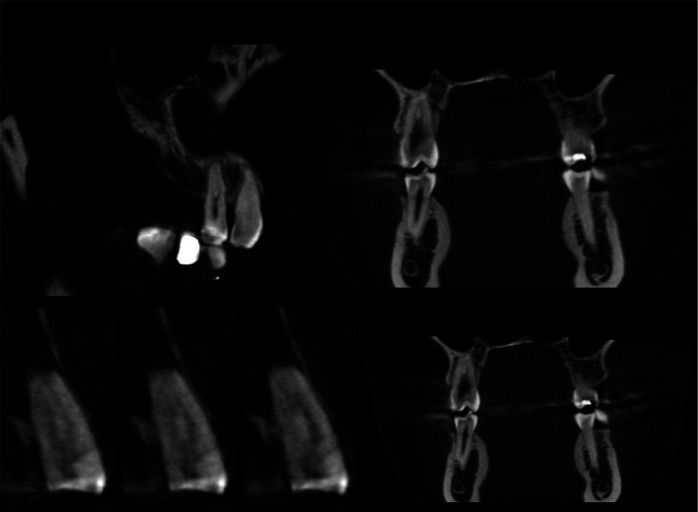
The coronal and sagittal CBCT (Cone-Beam Computed Tomography) view of secondary maxillary premolar with single root

#### Type of roots

As a general finding, the most common canal configuration for the maxillary second premolar was type I (n= 252; 63%), followed by type II (n= 78; 19.5%) and type IV (n = 57; 14.25%).

#### Number of apical foramens

The second maxillary premolar with 1, 2 or 3 apical foramens were found in almost (n= 333; 83.25%), (n= 63; 15.75%) and (n= 4; 1%) cases respectively. Among single-rooted maxillary second premolars, 333 teeth (91.48%) had one foramen in each root and 31 (8.52%) had two foramens. Almost all the two-rooted maxillary second premolars had one foramen in each root. Only two cases with two foramens in the buccal root and one foramen in the palatal root were found. In all three-rooted first premolars, there was one foramen in each root. In Table 2 , the number of roots is classified according to gender. In both maxillary first and second premolars, there was a significant relationship between the number of roots and sex (*p*= 0.002 and *p*= 0.001 respectively). Single-rooted teeth were dominant in females and two-rooted were prevailing in males.

In  Table 3, the number of maxillary premolar roots has been classified according to their position. According to this table, there was no significant relationship between root number and tooth position in maxillary first premolar (*p*= 0.754) and second premolar (*p*= 0.277). Regarding the interexaminer agreement, the Cohen kappa values after the training session were 0.993 and 0.995 for the first and second assessments, respectively.

**Table 2:** Classification of maxillary premolars according to gender and number of roots.

Tooth position	Single root (%)	Two roots (%)	Three roots (%)	Total
Maxillary 1^st^ premolars				
Female	158 (56.83)	116 (41.73)	4 (1.44)	278
Male	74 (40.22)	106 (57.61)	4 (2.17)	184
Total	232 (50.22)	222 (48.05)	8 (1.73)	462
Maxillary 2^nd^ premolars				
Female	227 (95.38)	10 (4.20)	1 (0.42)	238
Male	137 (84.57)	24 (14.81)	1 (0.62)	162
Total	364 (91.00)	34 (8.50)	2 (0.50)	400

**Table 3:** Classification of maxillary premolars according to tooth position and number of roots.

Tooth Position	Single root (%)	Two roots (%)	Three roots (%)	Total
Maxillary 1^st^ premolar				
Right	112 (48.48)	115 (49.78)	4 (1.73)	231
Left	120 (51.95)	107 (46.32)	4 (1.73)	231
Total	232 (50.22)	222 (48.05)	8 (1.73)	462
Maxillary 2^nd^ premolar				
Right	179 (89.5)	19 (9.5)	2 (1.0)	200
Left	185 (92.5)	15 (7.5)	0 (0)	200
Total	364 (91.0)	34 (8.5)	2 (0.5)	400

The overall Cohen kappa value for intraexaminer agreement was 0.998. In brief, there was a very good intraexaminer and interexaminer agreement.

## Discussion

One of the most common causes of failure in root canal treatment, especially in premolars, is missed canals due to the inadequate knowledge of the specific morphologies in this group of teeth [ 2 ]. This study was designed to evaluate the prevalence of extra roots and canals in maxillary premolars among an Iranian subpopulation using CBCT. Of various methods of studying the canal morphology, CBCT has been selected in the present study. Neelakantan *et al.* [ 19 ] revealed that CBCT can show the root canal system with the precision of staining and clearing technique and more accurately than the conventional radiography. Moreover, other studies also found that CBCT was more accurate in detecting additional canals than intra-oral radiography [ 20 , 21 , 33 ].

In addition to the accuracy of this method, CBCT is a non-invasive technique, which, unlike in-vitro methods, does not require extracted teeth and thus, can provide better information concerning the patients’ gender, age, and even teeth symmetry, which is not possible to obtain by other laboratory methods [ 19 ]. Variations in the root and canal morphology exist among various populations; the present study provides a report on the root morphology and canal configuration of maxillary premolars in Iranian subpopulation. Compared to other studies on Iranian populations, the present study was the first to evaluate both maxillary premolars using CBCT. 

In studies on other Iranian subpopulations, like an in-vitro study by Ketabi et al. in Isfahan [ 28 ], single rooted maxillary first premolars were more prevalent.

In addition, Tofangchiha et al. [ 27 ] in Ghazvin showed that single rooted maxillary first premolars were more prevalent. Similar to our study, Tofangchiha et al. also used CBCT in their research. However, in a study by Ghate et al. [ 29 ] two-rooted maxillary first premolars were more prevalent.

Similar to our study, in studies on other nationalities, Tian et al. [ 11 ] and Celikten et al. [ 34 ] showed that single rooted maxillary first premolars were more prevalent. However, in contrast to our findings, Abella et al. [ 7 ] and Alqedairi et al. [ 35 ] reported a more prevalent maxillary first premolar with two roots. In this study, the prevalence of two-rooted maxillary first premolars was (n=222; 48.05%). Only one (0.45%) of the two-rooted first premolars showed three canals, with two canals being in the buccal root and one canal located in the palatal root. This finding was not observed in recent morphological studies using CBCT as they reported a single canal in each root in the two-rooted and three-rooted first premolars [ 7 , 11 , 34 , 36 ]. Only Nazeer et al. [ 37 ] have observed three canals in the single-rooted and two-rooted premolars in its population.

Lipski et al. [ 38 ] first accredited the term “ridiculous” to the three-rooted first premolars, which is a characteristic of the Caucasian race. In the present study, the prevalence of maxillary first premolars with three distinct roots and three canals was observed to be 1.74%, which is similar to the findings of Ketabi et al. [ 28 ] who also reported a prevalence of 1.85% for the three-rooted maxillary first premolars in their in-vitro study. This finding is also in line with the reported values ​​in the literature and those studies using the CBCT method (0-6%) [ 7 , 11 , 34 , 37 , 39 ]. Only Nazeer *et al.* [ 37] and Yang *et al.* [ 40] did not observe three-rooted maxillary first premolars in their studies. In general, single rooted maxillary second premolar is the most common type of this group of teeth reported in recent studies ( Tables 4 and  5). Similarly, in the present research, the prevalence of second premolars with a single root was observed to be (n= 364; 91%). In our study, two cases of maxillary first premolars with two roots were observed to have three canals. This finding has not been previously reported in other studies. Moreover, in this study, the prevalence of three roots with three distinct canals was reported to be (n= 2, 0.5%), which was consistent with the range reported in previous studies (0-1%) [ 40]. 

**Table 4:** Root forms in maxillary first and second premolars in previous studies on Iranian subpopulations and the present study.

	Author (year)	Population (sample size)	One root (%)	Two roots (%)	Three roots (%)	Most common canal configuration (%)	Method	3 roots with 3 canals
Maxillary first premolar	Ketabi *et al.* (2008)	Isfahan (n=162)	66.6	31.48	1.85	-	*In vitro*In vitro sectioning	1.85
	Ghate *et al.* (2013)	Yazd (n=180)	19.5	79.4	1.1	IV (60.0)	*In vitro*In vitro endodontic access	1.1
	Tofangchiha *et al.* (2018)	Ghazvin (n=106)	57.8	41.4	0.9	II (56.7)	CBCT	0.9
	Present Study	Shiraz (n=462)	50.2	48.0	1.7	IV (71.6)	CBCT	1.7
Maxillary second premolar	Partovi *et al.* (2005)	Mazandaran (n=100)	98.0	2.0	-	II (48.0)	Staining	-
	Present Study	Shiraz (n=400)	91	8.5	0.5	I (63%)	CBCT	0.5

**Table 5:** Comparison of root forms in maxillary first and second premolars (based on Vertucci classification) in previous studies. on CBCT and those in this study

	Author (year)	Population (sample size)	One root (%)	Two roots (%)	Three roots (%)	Most common canal configuration (%)
Maxillary first premolar	Tian *et al.* (2012)	Chinese (300)	66.0	33.0	1.0	IV (51.0)
	Ok *et al.* (2014)	Turkish(1379)	-	-	-	IV (76.9)
	Felsypremila *et al.* (2015)	Indian (418)	48.4	51.2	-	IV (58.0)
	Abella *et al.* (2015)	Spanish (430)	46.0	51.4	2.6	IV (52.8)
	Celikten *et al.* (2016)	Turkish Cypriot (436)	53.7	44.8	0.9	IV (76.8)
	Shi *et al.* (2017)	Chinese (521)	60.8	37.8	1.3	IV (52.0)
	Alqedairi *et al.* (2018)	Saudi (334)	23.7	75.1	1.2	IV (70.6)
	Nazeer *et al.* (2018)	Pakistani (114)	31.5	68.5	-	I (68.0)
	Popovic *et al.* (2018)	Serbian (129)	42.6	53.5	3.9	IV (58.9)
	Martins *et al.* (2018)	Portuguese (714)	48.7	49.1	2.1	IV (68.2)
	Present Study	Iranian (462)	50.2	48.0	1.7	IV (71.6)
Maxillary second premolar	Yang *et al.* (2014)	Chinese (392)	86.5	13.5	-	I (45.4)
	Ok *et al.* (2014)	Turkish (1301)	-	-	-	I (54.5)
	Felsypremila *et al.* (2015)	Indian (393)	90.6	9.4	-	I (55.1)
	Abella *et al.* (2015)	Spanish (374)	82.9	15.5	1.6	I (39.3)
	Celikten *et al.* (2016)	Turkish Cypriot (445)	91.9	7.6	0.4	I (49.4)
	Shi *et al.* (2017)	Chinese (517)	92.4	7.5	-	II (40.0)
	Alqedairi *et al.* (2018)	Saudi (318)	85.2	14.5	0.3	I (49.4)
	Nazeer *et al.* (2018)	Pakistani (115)	84.3	15.7	-	I (53.4)
	Popovic *et al.* (2018)	Serbian (109)	88.1	11.9	-	I (59.6)
	Martins *et al.* (2018)	Portuguese (618)	94.6	5.3	-	I (40.0)
	Present Study	Iranian (400)	91	8.5	0.5	I (63%)

Partovi *et al.* [ 41] reported no cases of three roots with three canals in their in vitro study. Nazeer *et al.* [ 37 ] also reported a prevalence of 0% for three-rooted maxillary second premolars in their study.

Awareness of the prevalence of different canal configurations can be effective in the success of root canal treatment. In the present study, the highest variation in the type and configuration of the canals was observed for the single-rooted first and second premolars. Types IV and I, which are relatively easy to diagnose and treat, were the most commonly observed canal configurations among the maxillary first and second premolars respectively. Similar findings have been observed in most of the studies using CBCT ( Table 5). The following most common types detected in our study were types II and I in the maxillary first premolar, and types II, and IV in the maxillary second premolar.

The two-rooted maxillary first premolars showed almost only type IV canal configuration (n=221;99.55 %) with just one case of type VIII (n= 1; 0.45%). In the three-rooted maxillary first premolars, the only canal configuration was type VIII (n= 8; 100%). This type was not observed in single-rooted teeth. Amid maxillary second premolars, the single-rooted teeth had the highest variation in canal types. Type I had the highest prevalence in single-rooted second premolars (n=252; 69, 23%). 

Two-rooted second premolars had almost only type IV (n= 32; 94.12%) with two cases of type VIII (n = 2; 5.88%) canal configuration. In the three-rooted second premolars, only type VIII was detected (n= 2;100%).

In this study, type VII was the only configuration, which was not found in any of the first or second premolars. Likewise, Ok *et al.* [ 39 ] did not observe a type VII configuration in their study using CBCT. Caliskan *et al.* [ 42 ] and Awawdeh *et al.* [ 43 ] also reported no cases of this type of canal configuration in their in-vitro studies. Similarly, Pineda and Kuttler [ 44 ] did not detect any cases of types III, VI, and VII in their research.

Furthermore, in the present study, type VIII was observed in both two-rooted and three-rooted premolars. In a study by Nazeer *et al.* [ 37 ], type VIII was reported in single-rooted and two-rooted premolars. These differences observed in the number of roots and canals may be due to the different method of analysis, the sample size, and the geographically diverse populations.

Felsyprenila *et al.* [ 45] has reported a bilateral symmetry in 81.5% of the maxillary first and second premolars. In this study, all the first and second premolars were selected bilaterally. Therefore, it seems that there is a bilateral symmetry in the number of roots in the first and second premolars of maxilla. Only in the maxillary second premolar, no cases of three-rooted teeth were seen on the left side; however, this was not statistically significant. Like previous researches [ 7 , 11 , 34 , 36 ], no significant correlation was found between the number of roots and tooth positions in our study (*p*= 0.524).

Gender is one of the other factors, which can affect the morphology and number of roots [ 7 , 23 , 24 ]. In our study, a significant relationship was shown between sex and root number in both premolars (*p*< 0.001), as the single-rooted teeth were dominant in females and two-rooted premolars were more common in men. 

In line with our study, Martin *et al.* [ 24 ] and Ok *et al.* [ 39 ] showed that females had significantly lower number of roots with less root canal system complexity compared to males. Celikten *et al.* [ 34 ], Alqedairi *et al.* [ 35], and Popovic *et al.* [ 36 ] also found less root canal system complexity in females; however, these findings were not significant. In contrast to our study, Abella *et al.* [ 7 ] reported a similar number of roots between males and females. Considering the immensity of our country, the results of the present study cannot be applied to all Iranian population and it is suggested that further studies be conducted to investigate the number of roots and canal morphology in this group of teeth. Moreover, the relationship between tooth morphology with gender and tooth position needs further investigation. 

## Conclusion

This study can provide guidance to the root canals of maxillary premolar teeth for Iranian subpopulation leading to more optimal diagnosis and treatment planning for the endodontists. According to the findings, the complexity of root canal system and the number of roots were less observed in females compared to males.
